# The impact of neoadjuvant relugolix on multi-dimensional patient-reported fatigue

**DOI:** 10.3389/fonc.2024.1412786

**Published:** 2024-08-12

**Authors:** Jessica Y. Hsueh, Lindsey Gallagher, Min Ji Koh, Sarthak Shah, Malika Danner, Alan Zwart, Marilyn Ayoob, Deepak Kumar, Paul Leger, Nancy A. Dawson, Simeng Suy, Sean P. Collins

**Affiliations:** ^1^ Department of Radiation Medicine, MedStar Georgetown University Hospital, Washington, DC, United States; ^2^ Biotechnology Research Institute, North Carolina Central University, Durham, NC, United States; ^3^ Department of Oncology, Lombardi Comprehensive Cancer Center, MedStar Georgetown University Hospital, Washington, DC, United States

**Keywords:** prostate cancer, stereotactic body radiation therapy (SBRT), facit, relugolix, fatigue, quality of life, androgen deprivation therapy (ADT)

## Abstract

**Introduction:**

Androgen deprivation therapy has been shown to improve cancer control when combined with radiotherapy. Relugolix is an oral GnRH receptor antagonist that achieves rapid profound testosterone suppression, which may increase the perception and/or impact of fatigue. This study sought to evaluate neoadjuvant relugolix-induced fatigue in prostate cancer patients prior to the start of stereotactic body radiation therapy (SBRT).

**Methods:**

Relugolix was initiated at least two months before SBRT. The 13-item Functional Assessment of Chronic Illness Therapy-Fatigue (FACIT-F) questionnaire was collected at baseline and one hour prior to SBRT initiation. A five-point scale was used to score individual items. Overall scores range from 0-52 and individual item scores were converted to 0-100, with higher scores reflecting less fatigue. Five “experience” items explored self-perceptions of fatigue, and eight “impact” items sought to evaluate the effect of fatigue on daily activities. Items were evaluated for statistical significance (paired t-test, p < 0.05) and clinical significance (minimally important difference (MID); 0.5 standard deviation from baseline).

**Results:**

Between March 2021 to December 2023, 89 men were treated at Georgetown with neoadjuvant relugolix and SBRT. Mean age was 71 years (range: 49-87). Median initiation of relugolix was 4.5 months prior to SBRT (range: 2-14.2 months). 93% patients achieved castration (testosterone levels ≤ 50 ng/dL) and 85% patients achieved profound castration (testosterone levels ≤ 20 ng/dL). 87 patients completed the FACIT-F questionnaire, with an average overall score of 45.6 at baseline and 41.0 at SBRT initiation. This difference was statistically and clinically significant (p < 0.01, MID = 3.55). Patients experienced an increase in fatigue for 12 of 13 items, with statistically significant changes for 11 items. Three of five experience items showed a clinically significant increase in fatigue. Only two of eight impact items were clinically significant.

**Discussion:**

Our study shows that relugolix significantly increases fatigue, affecting multiple areas of life. While the fatigue does not appear to generally impact a patient’s ability to carry out normal activities, patients demonstrate frustration with being too tired for these activities. It is essential for clinicians to counsel prostate cancer patients on the impact of neoadjuvant relugolix on quality-of-life issues like fatigue.

## 1 Introduction

The National Comprehensive Cancer Network (NCCN) currently recommends radiation therapy (RT) in conjunction with androgen deprivation therapy (ADT) as the standard of care for unfavorable intermediate to high-risk prostate cancer (1). The addition of ADT to RT has been shown to improve overall survival when compared to RT alone (2, 3). Stereotactic body radiation therapy (SBRT), a newer more convenient RT option, has demonstrated efficacious biochemical control in prostate cancer with an excellent long-term safety profile (4). Recent data suggests that addition of ADT to SBRT for unfavorable prostate cancer may increase the probability of eliminating local disease and improving biochemical recurrence-free survival (5–7). Unfortunately, ADT remains underutilized due to bothersome symptoms such as fatigue (8).

There are many different ADT options, all with the common goal of suppressing testosterone production and therefore limiting prostate cancer progression. GnRH agonists such as leuprolide are the most commonly utilized. However, they cause an initial testosterone flare, which may worsen urinary retention and bone pain and causes a delay in castration (9). Relugolix is a novel oral GnRH receptor antagonist that has demonstrated superior sustained testosterone suppression in prostate cancer patients when compared to leuprolide (10). Because GnRH receptor antagonists do not induce a testosterone flare, relugolix can achieve earlier profound castration (testosterone ≤ 20 ng/dL) (10). In addition, relugolix is also associated with faster and more predictable testosterone recovery, which has important clinical implications for the management of side effects (11).

One of the most common side effects of prostate cancer treatment is fatigue. Referred to as cancer-related fatigue (CRF), it is defined by NCCN as a “distressing, persistent, subjective sense of physical, emotional, and/or cognitive tiredness or exhaustion related to cancer or cancer treatment that is not proportional to recent activity and interferes with usual functioning.” (12) A meta-analysis by Luo et al. estimated that CRF was prevalent in 40% of prostate cancer patients receiving RT, although a study by Dash et al. characterized CRF as a short-term effect of SBRT (13, 14). CRF is also frequently reported during ADT, with an estimated 42% prevalence (13). Studies have demonstrated that patients on ADT reported more severe and clinically meaningful fatigue at six and 12 months in comparison to prostate cancer patients who never received ADT (15). In the HERO trial, approximately 20% of patients who received relugolix or leuprolide experienced physician-reported fatigue, the majority of which was low grade (≤ grade 2) (10). The etiology of ADT-induced fatigue is unclear, although it is postulated that androgen deprivation can result in anemia because of testosterone’s stimulatory effects on erythropoiesis (16, 17).

Fatigue is a crucial quality of life (QoL) metric with important clinical and practical implications for patients. Baseline fatigue has emerged as a useful prognostic factor, with studies demonstrating that severe fatigue is significantly associated with increased mortality (18, 19). Similarly, a large meta-analysis of 43 oncologic clinical trials highlighted baseline fatigue as a strong predictor of overall survival (20). Among prostate cancer patients treated with SBRT, those encountering significant fatigue demonstrated inferior survival compared to counterparts reporting less pronounced fatigue (21). Beyond survival implications, CRF can cause major distress for patients and impact their ability to carry out daily activities. Despite ADT being a viable and effective treatment option for prostate cancer, few studies have elucidated on ADT and fatigue. Our investigation aims to examine how neoadjuvant relugolix prior to SBRT impacts patient-reported fatigue in prostate cancer patients.

## 2 Materials and methods

We conducted a prospective investigation of intermediate to high-risk prostate cancer patients treated with neoadjuvant relugolix and SBRT at MedStar Georgetown University Hospital (IRB 12-1775). We reviewed medical records to collect demographic information on age and race. We also extracted clinical information on body mass index (BMI), Gleason score, prostate volume, prostate-specific antigen (PSA), and testosterone levels after receiving relugolix. Risk groups were established using the D’Amico classification. The Charlson Comorbidity Index (CCI) was calculated to assess patients’ comorbidities at baseline.

### 2.1 Drug treatment

Neoadjuvant relugolix was initiated at least two months prior to SBRT. A 360 mg loading dose was given on the first day, with 120 mg oral doses taken daily at approximately the same time each day. Patients were counseled on common side effects, including fatigue.

### 2.2 Fatigue assessment

To assess self-reported patient fatigue, the validated Functional Assessment of Chronic Illness Therapy-Fatigue (FACIT-F) questionnaire was collected at initial consultation and one hour prior to SBRT initiation after several months of relugolix (22, 23). 13 individual items were scored on a five-point Likert scale: 0 (not at all), 1 (a little bit), 2 (somewhat), 3 (quite a bit), and 4 (very much) (24). The recall period, defined as the time patients are asked to consider when answering the questions, was seven days. Reversals were applied to negatively worded questions, and individual scores were summed to constitute a total possible overall score of 52, with higher scores reflecting less fatigue and better QoL (25).

**Figure 1:**
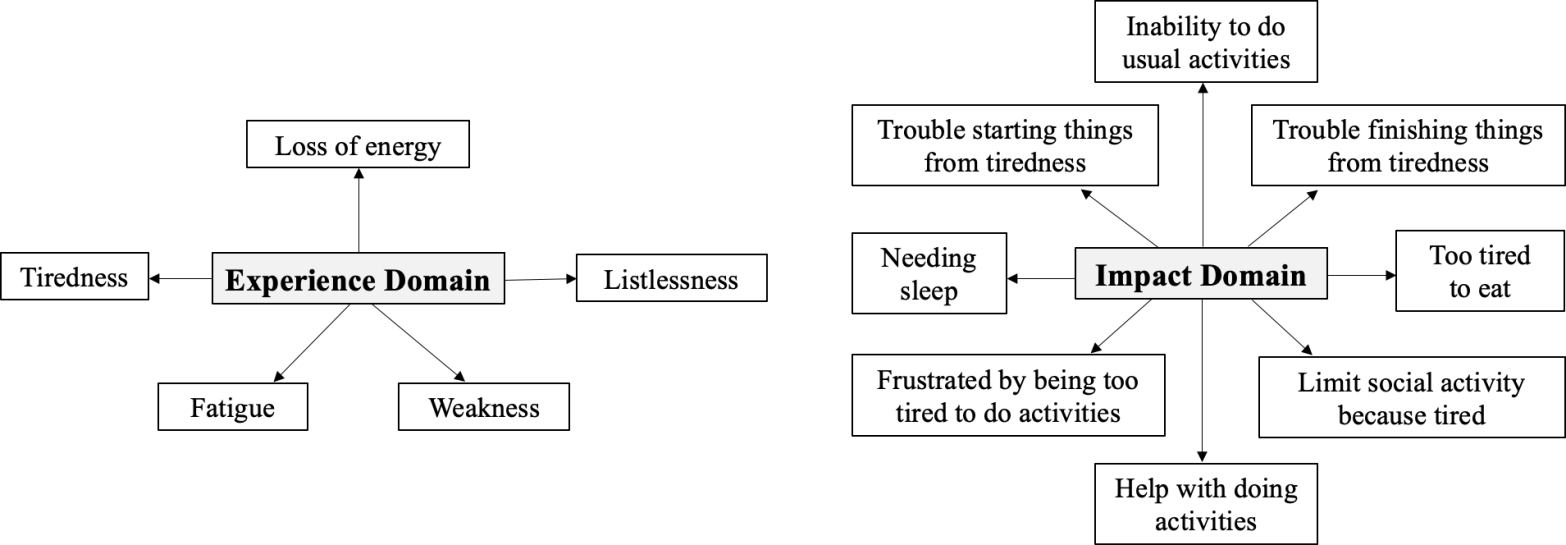
Diagram of the experience and impact domains for FACIT-F.

When conducting analysis for individual items, scores were transformed from 0 to 100. To capture the multidimensionality of fatigue, the FACIT-F questionnaire was divided into “impact” and “experience” domains (**Figure 1**) based on a paper by Cella et al. that explored how impact and experience were two conceptually different aspects of fatigue (26, 27). Five items that asked about self-perceptions of fatigue (fatigue, weakness, listlessness, tiredness, loss of energy) were classified as “experience.” The other eight items questioned how fatigue impacted daily activities (trouble starting things, trouble finishing things from tiredness, inability to do usual activities, needing sleep, being too tired to eat, requiring help for activities, experiencing frustration from being too tired to do activities, and limiting social activity) and were deemed as “impact.” Statistical significance was calculated by the paired t-test (p < 0.05). Clinical significance was evaluated by the minimally important difference (MID), defined as 0.5 standard deviation from baseline.

## 3 Results

### 3.1 Patient demographics and characteristics

Between March 2021 to December 2023, 89 men were treated at Georgetown with neoadjuvant relugolix and SBRT (**Table 1**). Mean age was 71 years (range: 49-87), with only 7% patients below 60 years old. 53% of patients identified as white, with 34% black patients in our study. 25% were obese with a BMI ≥30 kg/m2. 81% of patients were considered intermediate risk, while 18% patients had high risk prostate cancer. The median baseline CCI score for our patient cohort was 0, with only 11% patients having a CCI score above 3. Median initiation of relugolix was 4.5 months prior to SBRT (range: 2-14.2 months). 93% patients achieved effective testosterone castration, with testosterone levels below 50 ng/dL, and 85% patients achieved profound testosterone castration, with testosterone levels below 20 ng/dL (**Table 2**).

**Table 1:** Demographic and clinical characteristics.

Characteristics	No (%) (n = 89)
Age at baseline (y), Mean ± SD	71±7.8
<60 60-69 70-79 >80	6 (7) 35 (39) 38 (43) 10 (11)
Race	
White Black Other	49 (53) 30 (34) 10 (11)
Gleason Score	
3 + 3 = 6 3 + 4 = 7 4 + 3 = 7 4 + 4 = 8 4 + 5 = 9	6 (7) 21 (24) 48 (54) 13 (15) 1 (1)
Risk Group	
Intermediate High Recurrent	72 (81) 16 (18) 1 (1)
Charlson Comorbidity Index, Median (IQR)	0 (0-1)
None Mild (1-2) Moderate (3-4) Severe (>5)	47 (53) 32 (36) 6 (7) 4 (4)
BMI (kg/m2), Median (IQR)	27 (25-30)
<18.5 18.5-24.9 25-29.9 30-34.9 35-39.9 40-44.9	0 23 (27) 41 (48) 15 (18) 5 (6) 1 (1) *(n = 85)*
Prostate Volume (cc), Median (IQR)	37 (28-49)
	*(n = 88)*
PSA (ng/ml), Median (IQR)	
Baseline At RT	8.3 (5.8-13) 0.84 (0.3-5.2)

**Table 2:** Testosterone levels at baseline.

Characteristics	No (%) (n = 89)
Effective Castration (Testosterone ≤ 50ng/dL)	
Yes No	83 (93) 6 (7)
Profound Castration (Testosterone ≤ 20ng/dL)	
Yes No	76 (85) 13 (15)

Effective castration was defined as testosterone levels ≤50 ng/dL, while profound castration was defined as testosterone levels ≤20 ng/dL.

### 3.2 FACIT-F responses

**Figure 2:**
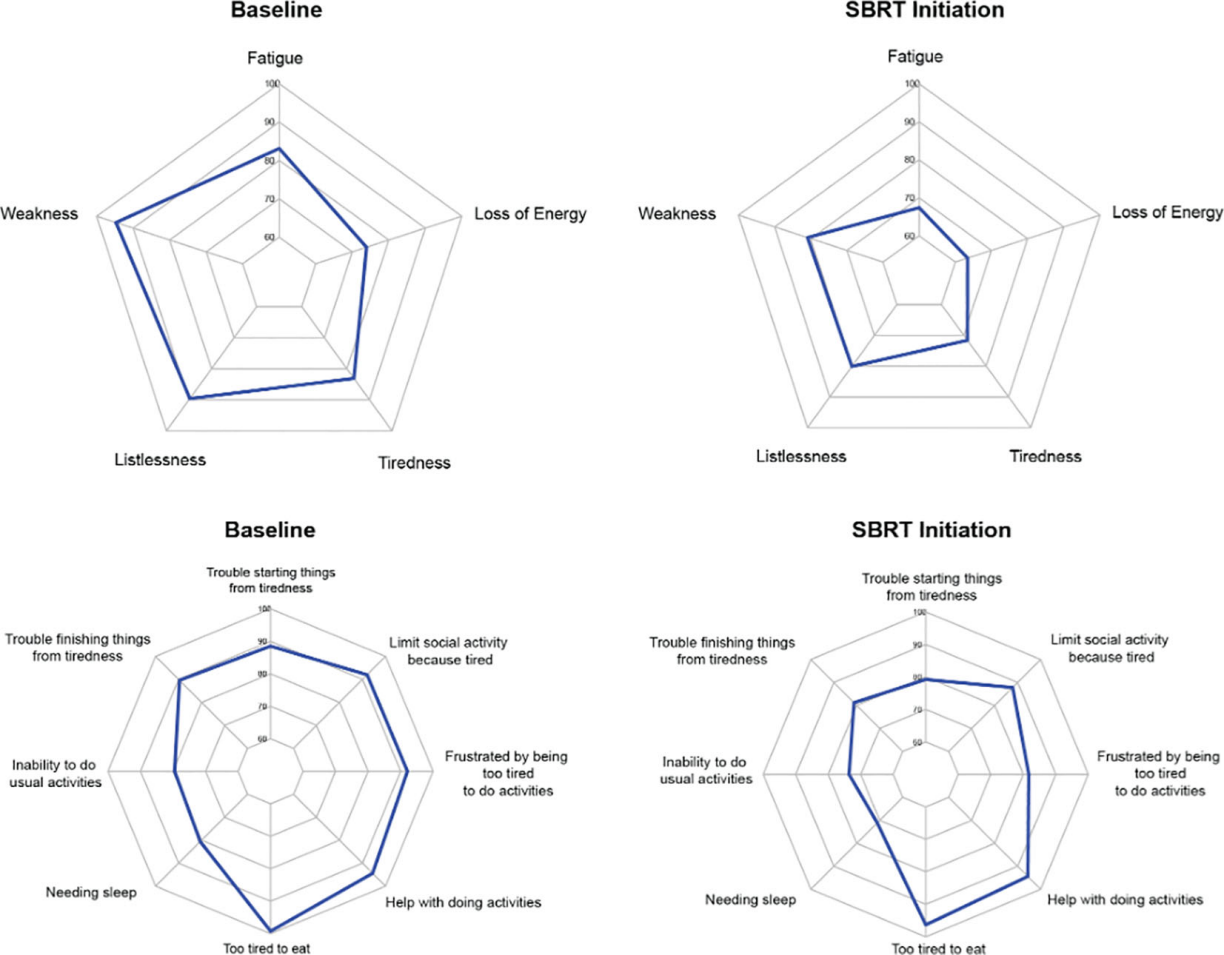
Radar plots showing the distribution of individual FACIT-F items at baseline and SBRT initiation. Individual item scores range from 0-100, with higher scores reflecting better quality of life. Points further from the center indicate a higher quality of life. The top row depicts radar plots for experience, while the bottom row depicts radar plots for impact.

The average overall scores were 45.6 at baseline and 41.0 after several months of relugolix (**Table 3**). This increase in fatigue was both statistically and clinically significant (p < 0.01, MID = 3.55). For the five items classified as “experience,” patients were found to report increased fatigue (83.1 to 67.4, MID = 12.03), weakness (94.7 to 80.9, MID = 8.10), listlessness (89.6 to 80.1, MID = 10.96), tiredness (83.1 to 71.6, MID = 11.1), and loss of energy (73.9 to 63.4, MID = 11.04) after relugolix (**Figure 2**). These changes were all statistically significant, although only three items- fatigue, weakness, and tiredness- were found to be clinically significant.

**Table 3:** FACIT-F scores at baseline and SBRT initiation for each item.

	Baseline (n = 87)	SBRT initiation (n = 87)	Statistical Significance (p-value)	Clinical Significance
**Overall Score**	45.6 ± 7.1 *(n = 85)*	41.0 ±10.0 *(n = 85)*	<0.01	Yes
Experience Items				
Fatigue	83.1 ± 24.1	67.4± 30.2	<0.01	Yes
Weakness	94.7 ± 16.2	80.9± 26.4	<0.01	Yes
Listlessness	89.6 ± 21.9	80.1± 28.0	<0.01	No
Tiredness	83.1 ±22.2	71.6 ± 27.5	<0.01	Yes
Loss of energy	73.9 ± 22.1 *(n = 86)*	63.4 ± 25.7 *(n = 86)*	<0.01	No
Impact Items				
Trouble starting things from tiredness	88.5± 20.7	79.2 ±25.9	<0.01	No
Trouble finishing things from tiredness	89.6 ±19.5	81.2 ±25.6	<0.01	No
Inability to do usual activities	79.5 ±25.7	73.6± 23.9	0.04	No
Needing sleep	80.6± 20.2	71.1 ±26.9	<0.01	No
Too tired to eat	99.2 ±4.54	96.3± 12.8	0.03	Yes
Help with doing activities	94.4 ± 15.4	94.4± 13.5	0.83	No
Frustrated by being too tired to do activities	92.1 ±17.5	81.7± 26.3	<0.01	Yes
Limit social activity because tired	92.0 ± 18.0 *(n = 86)*	87.8 ± 22.1 *(n = 86)*	0.06	No

Out of eight “impact” items, six items were considered statistically significant. However, only two items were considered clinically significant. Trouble starting things from tiredness (88.5 to 79.2, MID = 10.33), trouble finishing things from tiredness (89.6 to 81.2, MID = 9.76), needing sleep (80.6 to 71.1, MID = 10.10), inability to do usual activities (79.5 to 73.6, MID = 12.86), being too tired to eat (99.2 to 96.3, MID = 2.27), and frustration from being too tired to do activities (92.1 to 81.7, MID = 8.76) were all statistically significant. Only being frustrated by tiredness and being too tired to eat were clinically significant. The other two items, needing help with activities (94.4 to 94.4, MID = 7.71) and limiting social activity from tiredness (92.0 to 87.8, MID = 9.00), demonstrated neither clinically nor statistically significant changes.

## 4 Discussion

Our study is the first to demonstrate the effects of neoadjuvant relugolix on fatigue perception and disability in prostate cancer patients. FACIT-F has been validated to differentiate between fatigue experienced by cancer patients versus fatigue in the general population (28). The level of baseline fatigue in our study (45.6) is similar to what has been previously reported in the general US population (43.6) (28). After several months of relugolix, our patients’ average overall fatigue (41.0) is similar to what has been shown in non-anemic cancer patients (40.0) (28). We found that this increase in fatigue with relugolix was statistically and clinically significant. While a FACIT-F score of 41.0 indicates mild fatigue, our findings are consistent with previous reports of relugolix-induced fatigue. In the HERO trial, approximately 99% of fatigue experienced by patients on relugolix was classified as Grade 1 (relieved by rest) or 2 (not relieved by rest, limiting instrumental activities of daily living) (10).

Our study also represents an important step towards characterizing the multidimensionality of fatigue as a result of prostate cancer treatment. After relugolix, patients reported greater fatigue for almost all FACIT-F items. We observed statistically significant changes for 11 out of 13 items and clinically significant changes for five items. It appears that patients were mostly affected by their self-perceptions of fatigue, with clinically significant changes for three experience items: fatigue, weakness, and tiredness. The only clinically significant impact items were being too tired to eat and feeling frustrated about their tiredness. We postulate that the other items were not clinically significant because the fatigue was not significant enough to impact most daily activities. For example, needing help with activities was unchanged after relugolix; the increased fatigue was moderate enough to allow patients to continue carrying out their activities independently.

In line with our investigation, Cella et al. divided FACIT-F into experience and impact to investigate the multidimensionality of fatigue (26). The authors showed that while the overall FACIT-F score was still a reasonable endpoint and that experience and impact correlated strongly with each other, there were still significant differences in how patients reported the two domains. Our findings corroborate their conclusions that experience is more likely to be affected than impact because the effects on daily functioning are not yet pronounced (26). Another study demonstrated a correlation between self-reported fatigue and observed performance, with FACIT-F scores below 30 associated with increased difficulty in performing daily activities (27). Our investigation supports this finding, as our average overall FACIT-F scores were above 30 and we noted a lesser change in impact than experience. It is reassuring that fatigue was not more pronounced considering the high rates of profound castration (85%) in our population.

Current NCCN guidelines recommend screening for CRF, conducting a thorough physical exam and assessment, and managing fatigue based on clinical status (12). Typical management of ADT-induced fatigue includes physical activity, behavioral therapy, and nutrition counseling. In particular, exercise has been shown to significantly reduce CRF in patients with high levels of baseline fatigue (29). It is also critical to educate patients appropriately about the likelihood of experiencing CRF and its subsequent management. A small survey of prostate cancer patients on ADT found general dissatisfaction with their healthcare providers surrounding education and preparedness in managing their CRF (30). Survey participants reported that they often resorted to self-management strategies (30). Feeling frustrated about tiredness was one of two impact items that we found to be clinically and statistically significant. Even when patients at our institution were educated about fatigue and did not experience significant changes in their daily functioning, their frustration underscores the importance of counseling and providing effective tools to manage CRF.

Studies have suggested that CRF is greater in patients with significant medical comorbidities (31). A strength of our study is the inclusion of CCI, a metric to estimate comorbidity burden. Our median CCI was 0, indicating that the majority of our patients did not have any significant comorbidities that would have contributed to CRF. Only 11% of patients had a CCI above 3, meaning that their comorbidity burden was moderate or severe. Our patient cohort is also diverse, with 34% black patients included in our study.

Limitations to our study include a small sample size, given the recent FDA approval of relugolix in December 2020. Our investigation also had a relatively short follow-up period, with a median of 4.5 months of relugolix treatment. Because it is expected that fatigue would increase with longer ADT duration, future directions should characterize changes in fatigue over a longer period of time while also examining how fatigue changes with the rapid testosterone recovery experienced by patients after discontinuation of relugolix (15). It would also be beneficial to compare how patients treated with relugolix experience fatigue differently than patients treated with other ADT therapies.

## 5 Conclusions

Our study shows that neoadjuvant relugolix prior to SBRT significantly increases self-perceptions of fatigue, although the fatigue does not appear to generally impact patients’ abilities to carry out normal activities. Nevertheless, patients still demonstrate significant frustration with their fatigue, emphasizing the need to effectively counsel patients on QoL side effects. Limitations to our study include a small patient cohort and shorter follow-up period. Future directions for this investigation include comparing the effects of relugolix on fatigue to GnRH agonists, such as leuprolide.

## Data Availability

The raw data supporting the conclusions of this article will be made available by the authors, without undue reservation.
